# Central neuropeptide Y receptors are involved in 3^rd ^ventricular ghrelin induced alteration of colonic transit time in conscious fed rats

**DOI:** 10.1186/1471-230X-5-5

**Published:** 2005-02-18

**Authors:** Johannes J Tebbe, Clemens G Tebbe, Silke Mronga, Michael Ritter, Martin KH Schäfer

**Affiliations:** 1 Department of Internal Medicine, Division Gastroenterology – Endocrinology, Philipps Universität Marburg, 35033 Marburg, Germany; 2 Department of Internal Medicine, Division Cardiology, Philipps Universität Marburg, 35033 Marburg, Germany; 3 Department of Molecular Neuroscience, Institute of Anatomy and Cell Biology, Philipps Universität Marburg, 35033 Marburg, Germany

## Abstract

**Background:**

Feeding related peptides have been shown to be additionally involved in the central autonomic control of gastrointestinal functions. Recent studies have shown that ghrelin, a stomach-derived orexigenic peptide, is involved in the autonomic regulation of GI function besides feeding behavior. Pharmacological evidence indicates that ghrelin effects on food intake are mediated by neuropeptide Y in the central nervous system.

**Methods:**

In the present study we examine the role of ghrelin in the central autonomic control of GI motility using intracerobroventricular and IP microinjections in a freely moving conscious rat model. Further the hypothesis that a functional relationship between NPY and ghrelin within the CNS exists was addressed.

**Results:**

ICV injections of ghrelin (0.03 nmol, 0.3 nmol and 3.0 nmol/5 μl and saline controls) decreased the colonic transit time up to 43%. IP injections of ghrelin (0.3 nmol – 3.0 nmol kg^-1 ^BW and saline controls) decreased colonic transit time dose related. Central administration of the NPY_1 _receptor antagonist, BIBP-3226, prior to centrally or peripherally administration of ghrelin antagonized the ghrelin induced stimulation of colonic transit. On the contrary ICV-pretreatment with the NPY_2 _receptor antagonist, BIIE-0246, failed to modulate the ghrelin induced stimulation of colonic motility.

**Conclusion:**

The results suggest that ghrelin acts in the central nervous system to modulate gastrointestinal motor function utilizing NPY_1 _receptor dependent mechanisms.

## Background

The presence or absence of food in the gut stimulates the release of several regulatory peptides. These orexigenic (NPY, AGRP, ghrelin, MCH, Orexin-A, ...) and anorexigenic (CRF, CCK, CART, GLP-1, leptin, insulin, ...) peptides participating in the hypothalamic control of feeding behavior and satiety have been shown to be additionally involved in the autonomic control of gastointestinal (GI) functions like secretion and motility. For example fasted motor activity of the GI tract, e.g. the colon, is observed after intracerebroventricular (ICV) injection of neuropeptide Y whereas CRF ICV-treatment cause the disruption of fasted colonic motor activity [1]. Stomach-derived ghrelin is the first peripheral orexigenic peptide identified [2-6]. There is convincing evidence from several groups of investigators that ghrelin acts in the CNS and the periphery to simulate not only feeding but also GI function such as gastric acid secretion and gastric motility in rodents [7,9-11]. However, it is still unknown whether ghrelin is involved in the CNS control of other digestive functions besides gastric acid secretion and motility. Recent studies suggest that CNS-signaling by circulating ghrelin is mediated downstream by neurons of arcuate nucleus and the paraventricular nucleus of the hypothalamus, in particular, neurons expressing neuropeptide Y and agouti-related protein (AGRP) [12-14]. Furthermore it has been demonstrated that there is an anatomical interaction and functional relationship between ghrelin and neuropeptide Y. Using electrophysiological recordings Cowley et al have found that ghrelin stimulated the activity of arcuate NPYergic neurons and mimicked the effect of NPY in the paraventricular nucleus of the hypothalamus [15]. In addition ghrelin simulates food intake through hypothalamic NPY_1 _receptors [1,16,17]. Thus, the question came up "are NPY receptors involved in the ghrelin effect on GI function"? Among others, neuropeptide Y plays a role in the CNS control of gastrointestinal function [1,18]. NPY activates at least six receptor subtypes, NPY_1 _to NPY_6_. NPY binds preferentially with high affinity to Y_1 _and Y_2 _receptors, and there is evidence suggesting that these two receptor subtypes are involved in CNS regulation of digestive function by NPY action in arcuate nucleus and the paraventricular nucleus of the hypothalamus [18].

Taken together there is overwhelming evidence that ghrelin, beside its satiety modulatory capacity, is involved in the CNS control of digestive function of the upper gastrointestinal tract. In the CNS ghrelin and NPY, the most potent orexigenic neuropeptides known, are anatomical associated and functionally related. Moreover hypothalamic NPYergic neurons are downstream mediators of feeding related ghrelin action.

In the present study we scrutinize the hypothesis that central neuropeptide Y receptor activation is involved in the ghrelin induced modulation of gastrointestinal motility using a microinjection-model in conscious fed and freely moving rats.

## Methods

### Animals

All experimental components described were performed in accordance with the requirements of German legislation for the protection of animals and were licensed and supervised by the appropriate government body.

Male Sprague-Dawley rats with a mean body weight of 350 ± 50 g were maintained on a 12 : 12 h photoperiod. They were housed in colony cages under conditions of controlled humidity and temperature (22 ± 2°C) for at least 7 days prior to the surgical procedure. The animals were fed a standard rat diet (Altromin^®^, Lage, Germany) an tap water *ad libitum*. After surgical procedures, rats were housed individually. During experimental procedure the animals had continuous access to food and water.

### Drugs

Ghrelin (Bachem, Heidelberg, Germany) doses of 0.03 nmol (100 ng), 0.3 nmol (1 μg) and 3 nmol (10 μg)/5 μl were dissolved in 0.15 M sterile saline (B. Braun, Melsungen, Germany). The NPY_1 _receptor antagonist, BIBP-3226 (200 nmol/5 μl; Sigma-RBI, Natrick, MA, USA) [see Ref.: [19]] and the NPY_2 _receptor antagonist, BIIE-0246 (120 nmol/5 μl; Boehringer-Ingelheim, Biberach, Germany) [see Ref.: [21]] were dissolved in sterile 0.15 M saline. The NPY receptor antagonists were used in similar equipotent nanomolar concentrations. The used intracerebroventricular concentrations of the receptor antagonists were comparable with the ICV-dosages used by other groups in rodents [18,20]. Probes were aliquoted and frozen (-80°C). Fresh aliquots were thawed on each experimental day before injections. Any excess was discarded. In our hands nanomolar concentrations of BIBP-3226 and BIIE-0246 were effective in antagonization of NPY receptor subtypes without any side effects. In particular no central depressive effects or conspicuous behavior was observed after BIBP-3226 treatment [18].

### Cerebral cannulas

For surgical procedures, rats were anesthetized with a mixture of ketamine (75 mg kg^-1 ^i.p., Parke-Davis, Freiburg, Germany) and xylazine (5 mg kg^-1 ^i.p., Bayer AG, Leverkusen, Germany). Animals were positioned in a stereotactic apparatus (David Kopf Instruments, Tujunga, CA). The head was fixed in a nose-down-position (-3 mm) and the skull exposed. Then trepanation of the skullcap was performed according to coordinates obtained from Paxinos and Watson [22] (mm from bregma: anterior-posterior = -3.30; lateral = ± 0.0; dorsoventral = -3.8). According to these coordinates a 22-gauge guide cannula (Bilarney / Plastic one, Düsseldorf, Germany) was implanted into the third ventricle. The cannula was anchored by dental cement and stainless steel screws affixed to the skull. Dummy cannulas (28 G), extending 2 mm beyond the guide cannula tips, were inserted to prevent blockage. After cerebral surgery, animals were individually housed. The animals were allowed 4 days recovery after guide cannula surgeries before the abdominal surgical procedures were performed.

### Colonic catheter

This method was performed as described elsewhere [1]. Prior to all abdominal surgeries, the animals were food deprived overnight. Four days after cerebral surgery, rats were anaesthetized with a mixture of ketamine (75 mg kg^-1^) and xylazine (5 mg kg^-1^). After laparatomy a polyethylene microcatheter (inside diameter, 1.2 mm; outside diameter 1.7 mm; Becton Dickinson, New Jersy, USA) was chronically implanted into the proximal colon 1 cm distal from the caecocolonic junction. The catheter was fixed at the colonic wall by a purse-string suture and routed subcutaneously to the interscapular region, where it was exteriorized through the skin and secured. The animals were allowed 7 days recovery after abdominal surgeries before the beginning of habituation training sessions. Experiments were performed in fed, conscious rats.

### Intraperitoneal (IP) and intracerebroventricular (ICV) microinjection

The doses of ghrelin were calculated according to the lowest effective doses to stimulate food intake [see Ref.: [23]]. For IP injection a 1 ml syringe (Hamilton, Reno, NV, USA) was used. Ghrelin and vehicle were injected IP after central administration of vehicle or NPY receptor antagonist. For IP injections the low dose of ghrelin administered peripherally was 0.3 nmol kg^-1^/1 ml 0.15 M saline and the high dose of ghrelin was 3 nmol kg^-1^/1 ml saline. NPY receptor antagonists were injected ICV 15 min before ghrelin was given peripherally at doses of 200 nmol/rat (BIBP-3226) and 120 nmol/rat (BIIE-0246) respectively.

For ICV injections a 1 μl micro syringe (Hamilton, Reno, NV, USA), attached to a 32 G injection needle via a PE-50 tube-catheter was used. The stainless steel injection cannulas (32 G) were cut to protrude 2 mm beyond the tips of the guide cannulas. The conscious animals were gently restrained by hand, the injection needle was inserted through the guide cannula, and vehicle (5 μl 0.15 M saline) or NPY receptor antagonists (BIBP-3226 200 nmol/5 μl; BIIE-0246 120 nmol/5 μl) and ghrelin (0.03 nmol, 0.3 nmol or 3 nmol/5 μl), were consecutively injected ICV slowly over a 60 s period. We used a 15 min time interval between ICV injection of receptor antagonist or vehicle and ICV ghrelin administration. The injection needle was left in place for 2 min after injection to allow diffusion of the solution and to prevent back flow. Then dummy cannulas were reinserted into the guide cannulas. After the last experimental testing session, the rats were anesthetized and 5 μl of alcian blue 8GX were injected ICV.

### Colonic transit time measurement

Colonic transit time was calculated by using an enteral dye-marker. Trypan blue, a non-absorbable dye, was injected in 0.2 ml volume through the catheter positioned in the proximal colon and followed by a 0.2 ml saline flush immediately after the ICV or IP microinjection. Colonic transit time was evaluated as the time interval between dye injection and the discharge of the first blue pellet. Faecal pellet output was monitored continuously by a self-developed, automated observation system that mechanically registers the time of all bowel movements for 24 h. The device consists of a conveyor belt placed under the mesh bottom cage, which transports faecal pellets with defined velocity to a collector.

### Brain histology

The methods were performed as described in previous studies [17]. When experiments were completed, rats were anaesthetized with ketamine (75 mg kg ^-1 ^i.p.) and xylazine (5 mg kg ^-1 ^i.p.), and 0.05% alcian blue 8GX was microinjected intracerebroventricular under the same conditions as vehicle or peptide. The anaesthetized animals were transcardially perfused with phosphate buffered saline (PBS) buffer (0.1 M, pH 7.4) followed by Zamboni's fixative (2% formaldehyde and 2% picric acid in 0.1 M PBS buffer, pH 7.4). The brains were removed and cryoprotected in 25% sucrose. The site of injection was confirmed by inspection of intracerebroventricular dye distribution. Animals that received injections outside of the 3^rd ^ventricle were excluded from data analysis.

### Experimental design

#### Experiment I: Effect on colonic motorfunction of peripheral (IP) and central (ICV) ghrelin administration

The aim of the first experiment was to determine whether exogenous ghrelin would alter colonic motor function. Thus in experiment I, dose response effects of ghrelin in the cerebrospinal fluid (ICV) and the periphery (IP) on colonic transit time were examined. Ghrelin or saline as a vehicle was administered ICV or IP in conscious lightly restrained rats as previously described. For IP injection the low dose of ghrelin administered peripherally was 0.3 nmol kg^-1 ^BW and the high dose of ghrelin was 3.0 nmol kg^-1 ^BW. After injections, rats were subsequently returned to their home cages and maintained in a non-stressful environment to monitor colonic transit time. In order to minimize interindividual variation, and to reduce the number of animals needed to perform this study, animals were tested twice in this study. In randomized order, each rat received vehicle and a single dose of ghrelin or vehicle ICV or IP. The time interval between the experiments performed on the same animal was at least 4 days.

#### Experiment II: Effect of BIBP-3226 and BIIE-0246 pretreatment on centrally and peripherally injected ghrelin induced modulation of colonic transit

In experiment II the hypothesis that ghrelin acts at the CNS to modulate colonic motor function *via *a NPY receptor dependent pathway was addressed. Therefore, we determined if a pretreatment with selective NPY_1_- (BIBP-3226) and NPY_2 _(BIIE-0246) receptor antagonists administered into the cerebrospinal fluid would block the alterations of colonic motor activity induced by centrally and peripherally administered ghrelin. The animals were pretreated with the NPY-Y_1 _and -Y_2 _receptor antagonists, injected ICV or vehicle (0.15 M saline), 15 min prior to ICV or IP ghrelin injections. Thereafter colonic transit time was assessed as described above.

### Data analysis

The criterion used to include results in the data analysis of the ICV-injected group was the correct placement of the ICV cannulas.

Results are expressed as mean ± SEM. The data of all studies were analyzed by ANOVA and differences between groups were evaluated by the Student-Newmann-Keuls test. P < 0.05 was considered significant.

## Results

### Effect of peripherally (IP) and centrally (ICV) administered ghrelin on colonic motor function

**Figure 1:**
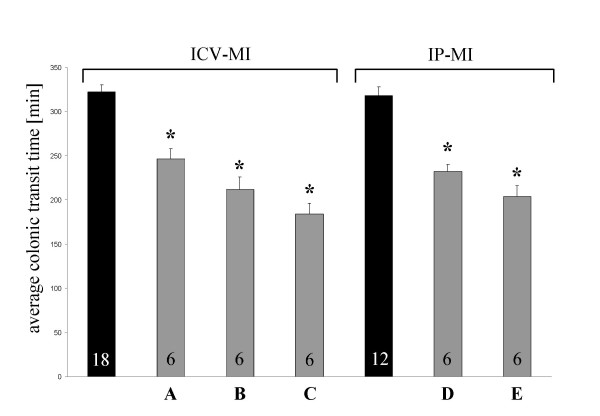
Effect of ghrelin injected into the 3^rd ^ventricle (ICV) and intraperitoneally (IP) on colonic transit time. Ghrelin injected ICV as well as IP induced a dosed-related stimulation of propulsive colonic motor activity. MI = microinjection The bars represent the mean ± SEM. * P < 0.05 vs. vehicle. ■ = vehicle-group; **A **= 0.03 nmol ghrelin /5 μl 0.15 M saline; **B **= 0.3 nmol ghrelin /5 μl 0.15 M saline; **C **= 3 nmol ghrelin /5 μl 0.15 M saline; **D **= 0.3 nmol ghrelin kg^-1 ^BW; **E **= 3.0 nmol ghrelin kg^-1 ^BW

In experiment I, dose response effect of peripherally and centrally administered ghrelin on colon transit time in fed and freely moving rats were examined. As demonstrated in Fig. 1, ghrelin injected into the cerebrospinal fluid (CSF) stimulates colonic transit time dose dependently. In rats microinjected with vehicle into the CSF or IP, the average colonic transit time was 322 ± 8 min. As demonstrated in Fig. 1, 0.03 nmol, 0.3 nmol and 3 nmol/5 μl ghrelin injected ICV dose-dependently decreased transit time by 24%, 34% and 43% respectively in conscious fed rats. Peripherally administered ghrelin accelerated transit time up to 36% (Fig. 1).

### Effect of ICV NPY receptor antagonist pretreatment on 3^rd ^ventricular and peripherally ghrelin induced stimulation of colonic transit

**Figure 2:**
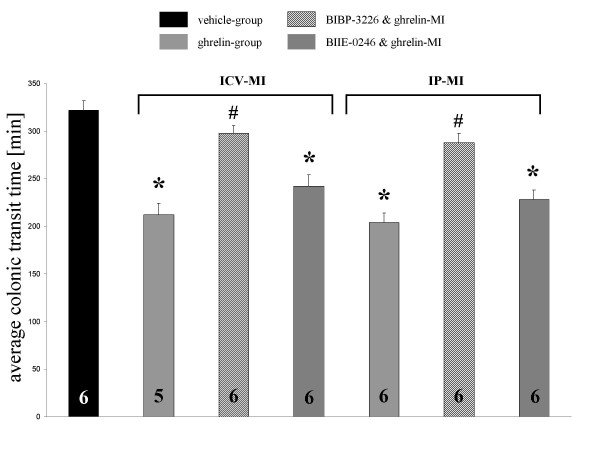
Effect of pretreatment with NPY receptor antagonists on fasted motor activity of the colon induced by centrally (ICV) and peripherally (IP) administered ghrelin. BIBP-3226, which is a selective NPY Y_1 _receptor antagonist, injected ICV antagonizes the stimulation of colonic transit induced by ghrelin injected in the same route and IP. The NPY Y_2 _receptor antagonist BIIE-2046 injected ICV fails to affect the stimulated colonic motoractivity induced by ICV or IP injection of ghrelin. MI = microinjection The bars represent the mean ± SEM. * P < 0.05 vs. vehicle-group; #P < 0.05 vs. ghrelin-group

The hypothesis that ghrelin acts in the brain to stimulate colonic transit *via *NPY receptor dependent mechanisms was addressed. As shown in Fig. 2, pre-treatment with BIBP-3226 (200 nmol) which is a selective NPY_1 _receptor antagonist 15 min prior to ICV application of 0.3 nmol ghrelin totally blocked the ghrelin induced effect on colonic transit. Application of BIBP-3226 into the CSF of the control group that was microinjected with vehicle, had no effect. Changes in colonic transit time induced by IP injection of ghrelin (3.0 nmol kg^-1 ^BW) were canceled by ICV injection of NPY_1 _receptor antagonist, BIBP-3226. (Fig.: 2). Pretreatment with the selective NPY_2 _receptor antagonist, BIIE-0246, failed to affect the ghrelin induced alteration of colonic transit time. (Fig.: 2)

## Discussion

The present experiments using a freely moving conscious rat model permit the measurement of colonic motility in rats in the physiological fed status. The results demonstrated that ghrelin given ICV and IP stimulates gastrointestinal motility indicated by shortened colonic transit time. In addition we found that the NPY type 1 receptor is primarily involved in the ghrelin induced modulation of fasted motor activity of the colon.

There is convincing evidence that the most effective appetite stimulating peptides, NPY and ghrelin, act in the CNS and the periphery to simulate not only feeding but also GI function such as gastric acid secretion and gastric motility [1,8-10]. Stomach derived ghrelin, first described in 1999 by Kojima et al., is the first peripheral orexigenic peptide identified [4]. Ghrelin was identified as endogenous ligand for the GH secretagogue receptor (GHS R) and a peripheral metabolic signal informing the brain about stomach nutrient load [17,24]. Physiological studies suggest a functional relationship of ghrelin and NPY within the brain. It has been demonstrated that peripherally (i.v.) and central (ICV) administered ghrelin increases the expression of the immediate early gene c-*fos*, a marker of neuronal activity, in the arcuate nucleus and the PVN in awake fed rats [25]. Furthermore, exogenous ghrelin increases mRNA levels for NPY into the arcuate nucleus and simulates food intake through hypothalamic NPY_1 _receptors [14,16,26]. Further Fujino et al. have demonstrated that ICV pretreatment with neuropeptide Y antiserum completely blocked ghrelin induced gastric and duodenal motoractivity [9]. Taken together these data suggest that there is an anatomical interaction and functional relationship between ghrelin and NPY within the brain. Six recognized subtypes of neuropeptide Y receptors have been described (NPY_1 _to NPY_6_). Two of these, NPY_1_- and NPY_2 _receptors, are found in high density in the hypothalamus. There is compelling evidence that, in particular, NPY_1 _and NPY_2 _receptors are involved in the CNS regulation of gastrointestinal function. [1,8,27] For this reason, we focused on neuropeptide Y_1 _and Y_2 _receptor pathways in the present study and did not investigate the role of neuropeptide Y receptor subtypes Y_4 _and Y_5 _which are also expressed in the hypothalamus and are also involved in the autonomic control of feeding behavior and GI function. In the present study pretreatment with the NPY_1 _receptor antagonist, BIBP-3226, blocked stimulation of colonic motility induced by systemic microinjection of exogenous ghrelin (ICV and IP). In our hands BIIE-2046, which is a selective antagonist of the NPY Y_2 _receptor, failed to affect the ghrelin induced induction of fasted motor activity. It was previously described that knocking out NPY significantly decreases ghrelin stimulated feeding [17,28]. In this context Fujino et al. have recently demonstrated that the ghrelin induced fasted gastroduodenal motor activity in rats is blocked by ICV injection of GHS-R antagonist as well as NPY antiserum [9]. The results presented by Fujino et al. also suggest that the vagal pathway may mediate the action of centrally administered ghrelin on gastroduodenal motility [9]. Thus we can speculate that central NPY pathways, e.g. centrally NPY receptor activation, are the primary downstream mediator of circulating ghrelin. This interpretation is consistent with neuroanatomical and physiological facts: Neuropeptide Y works at two sites, locally within the arcuate nucleus to inhibit POMC neuronal activity and at afferent-terminal sites, in particular the paraventricular nucleus of the hypothalamus. Guan et al. have shown that neuropeptide Y- and ghrelin like immunoreactive (LI) neurons within the arcuate nucleus could influence each other by complex synaptic transmissions [29]. Furthermore Cowley et al. have demonstrated that ghrelin stimulated the activity of arcuate neuropeptide Y-LI neurons and mimicked the effect of neuropeptide Y in the PVN [15]. Compelling evidence showed that NPY projections from the arcuate nucleus (ARC) to the PVN are involved in the CNS regulation of food intake and other physiological functions of the organism, e.g. digestive function, by neuroendocrine and autonomic pathways [17,18]. For example NPY released from ARC neurons activates NPY-Y_1 _receptors in the hypothalamus, e.g. the PVN, and results in the stimulation of GI motor function [18]. Furthermore arcuate NPYergic neurons have been thought to regulate feeding behavior by NPY receptor subtypes Y1 and Y5 in the PVN and adjacent areas [17]. Pretreatment with a Y1, but not other receptor antagonist markedly inhibited ghrelin-induced feeding, pointing to NPY receptor Y1 as one of the downstream pathways [9,17]. With regard to the characteristic physiological feature that peripheral ghrelin does not cross the blood-brain barrier in rodents it is important to note that the arcuate nucleus is the only hypothalamic structure located outside the brain-blood barrier [30]. Thus we can speculate that circulating ghrelin modulates gastrointestinal motility *via *activation of hypothalamic, in particular by using NPYergic pathways via activation of NPY-Y1 receptors, in the arcuate nucleus. This hypothesis is in good agreement with our observation that the effect of peripherally (IP) administered ghrelin on colonic motility is blocked by ICV pretreatment with the specific NPY_1 _receptor antagonist, BIBP-3226. The NPY_2 _receptor antagonist BIIE-0246 injected in the 3^rd ^ventricle at the equipotent dose as BIBP-3226 was not effective to antagonize the ghrelin effect on GI motility significantly. This data suggests that ghrelin unfolds a stimulatory effect on colonic motility primarily by acting on central NPY_1 _and not via NPY_2 _receptors. This interpretation is confirmed by the observation that Y_1 _receptors acts rather postsynaptically and the Y_2 _receptor rather presynaptically [31,32] The question of whether neuropeptide Y_4 _or Y_5 _receptors in the CNS are involved in the CNS control of gastrointestinal function should be examined in future studies.

## Conclusion

We hypothesize that circulating ghrelin exhibits its effect by activating hypthalamic neurons, in particular neurons in the arcuate nucleus bearing GHS- and NPY-Y1 receptors. Further this ghrelin induced neuronal activation leads to stimulation of GI motor function by activation of higher hypothalamic brain sites, e.g. activation of neuronal projections within the paraventricular nucleus of the hypothalamus. On the other hand it is possible that the site of action of circulating ghrelin is not the hypothalamus but other brain sites. In our model using 3^rd ^ventricular injection of ghrelin this could simply mean that the peptide gained access to the 4^th ^ventricle and reached further caudal brain sites, e.g. NTS, DVC and medulla oblongata. With respect to the distribution of GHS- and NPY receptors in the CNS this hypothesis is possible, but how ghrelin action on any of these brain sites would modulate digestive function is not known. This question should be examined in future studies

In summary, we presented evidence that ghrelin is involved in the CNS control of GI function. Apart from humoral pathways ghrelin acts into the CNS to control GI function by a mechanism of action involving neuropeptide Y1 receptor pathways. Further this study support the hypothesis giving by Chen et al. that ghrelin has an absolute requirement for neuropeptide Y pathways to unfold its physiological effects [28].

## List of abbreviations

AGRP agouti-related peptide

ARC arcuate nucleus

CART cocain- and amphetamine-regulated transcript

CCK cholecystokinin

CNS central nervous system

CSF cerebrospinal fluid

CRF corticotropin releasing factor

DVC dorsal motor nucleus of vagus

GHS-R growth hormone secretagogue receptor

GLP-1 glucagon like peptide-1

ICV intracerebroventricular

MCH melanocortin hormone

MI microinjection

NPY neuropeptide Y

NTS nucleus of the solitary tract

POMC proopiomelanocortin

PVN paraventricular nucleus of the hypothalamus

## Competing interests

The author(s) declare that they have no competing interests.

## Authors' contributions

JJT participated in the design and coordination of the study, performed the microinjection studies and drafted the manuscript. CGT was the surgeon in charge and participated in the animal experiments. M-KHS and SM were involved in the design and coordination of the study. MR participated in the analysis and interpretation of data and revised the manuscript critically.

## Pre-publication history

The pre-publication history for this paper can be accessed here:



## References

[B1] Mönnikes H, Tebbe J, Bauer C, Grote C, Arnold R (2000). Neuropeptide Y in the paraventricular nucleus of the hypothalamus stimulates colonic transit by peripheral cholinergic and central CRF pathways. Neurogastroenterol Moilt.

[B2] Asakawa A, Inui A, Kaga T, Yuzuriha H, Nagata T, Ueno N, Makino S, Fujimiya M, Niijima A, Fujino MA, Kasuga M (2001). Ghrelin is an appetite-stimulatory signal from stomach with structural resemblance to motilin. Gastroenterology.

[B3] Date Y, Kojima M, Hosoda H, Sawaguchi A, Mondal MS, Suganuma T, Matsukura S, Kangawa K, Nakazato M (2000). Ghrelin a novel growth hormone releasing acylated peptide, is synthesized in a distinct endocrine cell type in the gastrointestinal tract of rats and humans. Endocrinology.

[B4] Kojima M, Mosoda H, Date Y, Nakazato M, Matsuo H, Kangawa K (1999). Ghrelin is a growth-hormone-releasing acylated peptide from stomach. Nature.

[B5] Nakazato M, Murakami N, Date Y, Kojima M, Matsuo H, Kangawa K, Matsukura S (2001). A role for ghrelin in the central regulation of feeding. Nature.

[B6] Tomasetto C, Wendling C, Rio MC, Poitras P (2001). Identification of cDNA encoding motilin-related-peptide/ghrelin precursor from dog fundus. Peptides.

[B7] Date Y, Nakazato M, Murakami N, Kojima M, Kangawa K, Matsukura S (2001). Ghrelin acts in the central nervous system to stimulate gastric acid secretion. Biochem Biophys Res Commun.

[B8] Fujimiya M, Itoh E, Kihara N, Yamamoto I, Fujimura M, Inui A (2000). Neuropeptide Y induces fasted pattern of duodenal motility via Y2 receptors in conscious fed rats. Am J Physiol Gastrointest Liver Physiol.

[B9] Fujino K, Inui A, Asakawa A, Kihara N, Fujimura M, Fujimia M (2003). Ghrelin induces fasted motor activity of the gastrointestinal tract in conscious fed rats. J Physiol.

[B10] Masuda Y, Tanaka T, Inomata N, Ohnuma N, Tanaka S, Itoh Z, Hosoda H, Kojima M, Kangawa K (2000). Ghrelin stimulates gastric acid secretion and motility in rats. Biochem Biophys Res Commun.

[B11] Trudel L, Tomasetto C, Rio C, Bouin M, Plourde V, Eberling P, Poitras P (2002). Ghrelin/motilin-related peptide is a potent prokinetic to reverse gastric postoperative ileus in rat. Am J Physiol Gastrointest Liver Physiol.

[B12] Hewson AK, Tung LY, Connell DW, Tookman L, Dickson SL (2002). The rat arcuate nucleus integrates peripheral signals provided by leptin, insulin and a ghrelin mimetic. Diabetes.

[B13] Kamegai J, Tamura H, Shimizu T, Ishii H, Sugihara H, Wakabayashi I (2001). Chronic central infusion of ghrelin increases hypothalamic neuropeptide Y and aguoti-related protein mRNA levels and body weight in rats. Diabetes.

[B14] Seoane LM, Lopez M, Tovar S, Casanueva FF, Senaris R, Dieguez C (2003). Agouti-related peptide, neuropeptide Y, and somatostatin-producing neurons are targets for ghrelin actions in the rat hypothalamus. Endocrinology.

[B15] Cowley MA, Smith RG, Diano S, Tschop M, Pronchuk N, Grove KL, Strasburger CJ, Bidlingmaier M, Esterman M, Heiman ML, Garcia-Segura LM, Nillni EA, Mendez P, Low MJ, Sotonyi P, Friedman JM, Liu H, Pinto S, Colmers WF, Cone RD, Horvath TL (2003). The distribution and mechanism of action of ghrelin in the CNS demonstrates a novel hypothalamic circuit regulating energy homeostasis. Neuron.

[B16] Shintani M, Ogawa Y, Ebihara K, Abe AM, Miyagawa F, Takaya K, Hayashi T, Inoue G, Hosoda K, Kojima M, Kangawa K, Nakao K (2001). Ghrelin, an endogenous growth hormone secretagogue, is a novel orexigenic peptide that antagonizes leptin action through the activation of hypothalamic neuropeptide Y/Y1 receptor pathway. Diabetes.

[B17] Inui A (2001). Ghrelin: An orexigenic and somatotrophic signal from the stomac. Nat Rev Neurosci.

[B18] Tebbe JJ, Mronga S, Schäfer M-K-H, Rüter J, Kobelt P, Mönnikes H (2003). Stimulation of neurons in rat ARC inhibits gastric acid secretion via hypothalamic CRF 1/2- and NPY-Y1 receptors. Am J Physiol Gastrointest Liver Physiol.

[B19] Rudolf K, Eberlein W, Engel W, Wieland HA, Willim KD, Entzeroth M, Wienen W, Beck-Sickinger AG, Doods HN (1994). The first highly potent and selective non-peptide neuropeptide Y Y1 receptor antagonist: BIBP3226. Eur J Pharmacol.

[B20] Redrobe JP, Dumont Y, Fournier A, Quirion R (2002). The neuropeptide Y (NPY) Y1 receptor subtype mediates NPY-induced antidepressant-like activity in the mouse forced swimming test. Neuropsychopharmacology.

[B21] Doods H, Gaida W, Wieland HA, Dollinger H, Schnorrenberg G, Esser F, Engel W, Eberlein W, Rudolf K (1999). BIIE0246: a selective and high affinity neuropeptide Y Y(2) receptor antagonist. Eur J Pharmacol.

[B22] Paxinos G, Watson C (1997). The rat brain in stereotaxic coordinates. Academic Press, San Diego.

[B23] Olszewski PK, Grace MK, Billington CJ, Levine AS (2003). Hypothalamic paraventricular injections of ghrelin: effect on feeding and c-Fos immunoreactivity. Peptides.

[B24] Bednarek MA, Feighner SD, Pong SS, McKee KK, Hreniuk DL, Silva MV, Warren VA, Howard AD, Van Der Ploeg LH, Heck JV (2000). Structure-function studies on the new growth hormone-releasing peptide, ghrelin: minimal sequence of ghrelin necessary for activation of growth hormone secretagogue receptor 1a. J Med Chem.

[B25] Wang L, Saint P, Tache Y (2002). Peripheral ghrelin selectively increases Fos expression in neuropeptide Y-synthesizing neurons in mouse hypothalamic arcuate nucleus. Neurosci Lett.

[B26] Willesen MG, Kristensen P, Romer J (1999). Co-localization of growth hormone secretagogue receptor and NPY mRNA in the arcuate nucleus of the rat. Neuroendocrinology.

[B27] Tamira H, Kamegai J, Shimizu T, Ishii T, Sugihara H, Oikawa S (2002). Ghrelin stimulates GH but not food intake in arcuate nucleus ablated rats. Endocrinology.

[B28] Chen HY, Trumbauer ME, Chen AS, Weingarth DT, Adams JR, Frazier EG, Shen Z, Marsh DJ, Feighner SD, Guan XM, Ye Z, Nargund RP, Smith RG, Van der Ploeg LHT, Howard AD, Macneil DJ, Qian S (2004). Orexigenic action of peripheral ghrelin is mediated by neuropeptide Y and agouti-related protein. Endocrinology.

[B29] Guan JL, Wang QP, Kageyama H, Takenoya F, Kita T, Matsuoka T, Funahashi H, Shioda S (2003). Synaptic interactions between ghrelin- and neuropeptide Y-containing neurons in the rat arcuate nucleus. Peptides.

[B30] Banks WA, Tschop M, Robinson SM, Heiman ML (2002). Extent and direction of ghrelin transport across the blood-brain barriers is determined by its unique primary structure. J Pharmacol Exp Ther.

[B31] Colmers WF, Klapstein GJ, Fournier A, St. Pierre S, Treherne KA (1991). Presynaptic inhibition by neuropeptide Y in rat hippocampal slice in vitro is mediated by a Y2 receptor. Br J Pharmacol.

[B32] Wahlestedt C, Yanaihara N, Hakanson R (1986). Evidence for different pre- and post-junctional receptors for neuropeptide Y and related peptides. Regul Pept.

